# Design and Characterization of Langmuir–Blodgett Films Incorporating Europium Complexes and Nucleoside Derivatives for Cancer Therapeutic Applications

**DOI:** 10.1021/acsomega.5c06204

**Published:** 2025-09-12

**Authors:** Guilherme Coutinho Pereira, Kevin Figueiredo dos Santos, Jhon Fernando Berrío Escobar, Cristiano Giordani, Luciano Caseli, Celso Molina

**Affiliations:** † Department of Chemistry, 505146 Federal University of São Paulo, Diadema 09972-270, SP, Brazil; ‡ Institute of Physics, Faculty of Exact and Natural Sciences, 27983 Universidad de Antioquia, UdeA, Calle 70 No. 52-21, Medellín 050010, Colombia; § Grupo Productos Naturales Marinos, Faculty of Pharmaceutical and Food Sciences, Universidad de Antioquia, UdeA, Calle 70 No. 52-21, Medellín 050010, Colombia; ∥ Institución Educativa Ciudad Dorada-Secretaría de Educación de Armenia, Armenia 630002, Colombia

## Abstract

The design of multifunctional nanostructured materials with potential for both diagnostic and therapeutic use, the so-called theranostic platforms, is a promising strategy in cancer research. In this study, we report the fabrication and physicochemical characterization of Langmuir and Langmuir–Blodgett (LB) films composed of a triacylated uridine derivative (PNM2), previously described as a prodrug with anticancer potential, and the europium complex Eu­(tta)_3_(H_2_O)_2_, known for its luminescent and structural probe properties. Langmuir monolayers at the air–water interface exhibited high rigidity for PNM2, which was modulated upon incorporation of the europium complex. Surface pressure–area isotherms, surface potential data, and dilatational rheology confirmed a transition toward a more fluid-like yet stable film. PM-IRRAS and Brewster angle microscopy indicated uniform molecular organization and specific interactions between Eu^3^^+^ and the carbonyl groups of PNM2. Upon transfer to solid substrates, LB films preserved this organization and exhibited strong photoluminescence, with emission spectra confirming efficient energy transfer via the antenna effect. Lifetime and quantum efficiency measurements revealed reduced water coordination and enhanced radiative decay. These results demonstrate that PNM2–Eu­(tta)_3_(H_2_O)_2_ films are structurally robust and optically responsive systems, offering a promising foundation for the development of biofunctional interfaces with potential theranostic applications, including cancer sensing and localized drug delivery.

## Graphical abstract

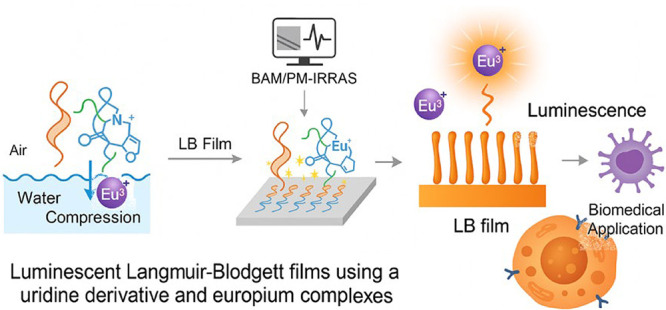

## Introduction

In recent years, the development of novel cancer therapies has increasingly focused on incorporating hydrophobic chains into the molecular structure of drugs. This strategy not only protects the drug from biological or enzymatic degradation due to the steric or electronic effects of the substituents but also enhances cell membrane permeability, potentially reducing the toxicity against normal cells. Notable among these drugs are nucleoside-based compounds, which have been used in cancer therapy since the 1960s.^1–3^ These compounds are known for their antiproliferative, cytotoxic, and cytostatic properties, with multiple mechanisms of action against tumorigenic cells.

Despite their promise, understanding the precise mechanisms of drug delivery, including where, when, and how these drugs reach the target cells, remains a significant challenge. The use of luminescent probes has emerged as a valuable strategy for developing biomarkers of pharmacological interest.^4–6^ Additionally, liposomes have been widely employed as drug delivery systems that involve interactions between drugs and lipids. Conveniently, these systems can incorporate luminescent probes as molecular markers.

In this context, Langmuir monolayer floating monomolecular films formed by amphiphilic molecules at liquid–gas interfaces have been extensively used as models for cellular membranes and other biological interfaces. These monolayers can effectively mimic the outer layer of cellular membranes.^7–9^ Moreover, when these monolayers are transferred to solid support as LB films, it enhances the potential for film characterization and their application in nano-optoelectronic devices, such as sensors and clinical probes.^10–12^

LB films containing europium complexes have garnered attention due to their ability to facilitate energy transfer through the antenna effect, which occurs due to the interaction between the ligand and the rare earth element. These luminescent films can be used as structural probes.^13–15^ For example, Singh et al. investigated the coordination properties of lanthanides with uridine groups of nucleotides in biological systems.^16^

Building on these concepts, this paper explores the interaction between an ester-type derivative of uridine, 3′, 4’, and 6’-tri stearoyl uridine (PNM2), and the europium complex Eu­(tta)_3_(H_2_O)_2_ (Figure 1), a compound chosen for its potential action against cancer cells. We prepared ultrathin films using Langmuir and LB techniques to investigate their capacity to form Langmuir monolayers, both pure and mixed with models for tumorigenic cells, and to recognize complementary bases in the aqueous subphase.^17,18^ This approach allows us to produce nanostructured films and conduct detailed investigations of their thermodynamic, morphological, and structural properties. The highly controlled molecular architectures of these films make them valuable for enhancing the physicochemical properties of nanostructured devices.

**Figure 1:**
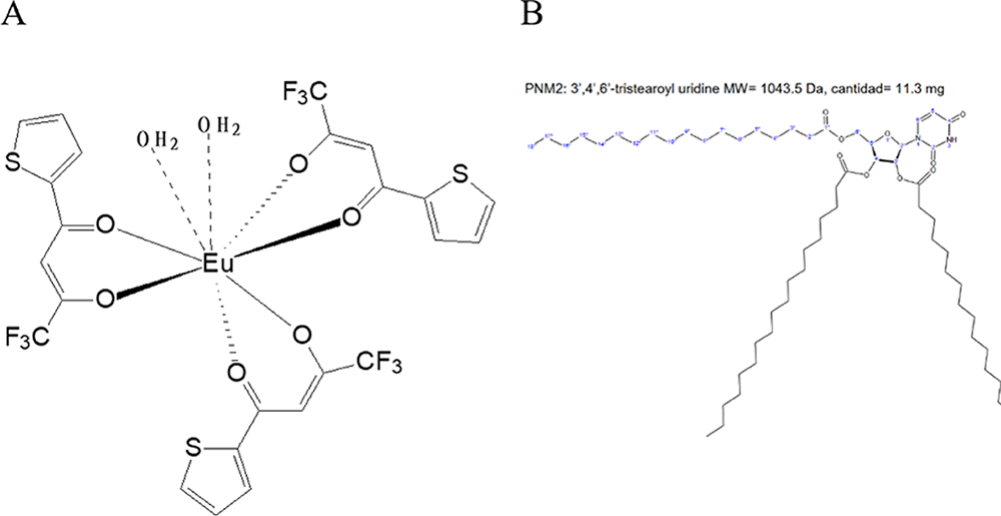
Eu­(tta)_3_(H_2_O)_2_ complex (A), and PNM2 (B).

Incorporating europium complex into bioinspired materials composed of lipids and nucleotides was the objective of this paper, because these materials can mimic biological environments, enhancing the biocompatibility and specificity of molecular probes. Europium’s unique luminescent properties allow for highly sensitive detection, making it an ideal candidate for imaging and tracking within biological systems.^19–21^ When integrated with alkylated nucleotide-based structures, these complexes can target cancerous cells more effectively by exploiting the natural affinity of these biomolecules for cell membranes and nucleic acids,^21^ offering potential advancements in cancer diagnostics and therapy.

## Materials and Methods

The prodrug PNM2 was synthesized following a previously described procedure.^22^ The complex [Eu­(tta)_3_(H_2_O)_2_] was synthesized by reacting 2-thenoyltrifluoroacetone (tta) with an aqueous solution of EuCl_3_·6H_2_O (also from Sigma-Aldrich), following an adapted procedure from the literature.^23^ Each compound was dissolved in chloroform to achieve an approximate concentration of 0.5 mg/mL. The chemical structures of the molecules are shown in Figure 1.

Langmuir films were prepared using a Langmuir trough (KSV-Instruments, model Mini) made of Teflon. The trough was first filled with water purified by the Milli-Q system (resistivity of 18.2 MΩ·cm). The solution of interest was then spread on the air–water interface by using a microsyringe. A waiting period of approximately 10 min was allowed for the chloroform to evaporate and for the monolayer to organize structurally. When mixed monolayers were composed of two or three components, the desired proportions of each solution were premixed before spreading to avoid aggregation due to poor interface spreading and to facilitate faster attainment of thermodynamic equilibrium. These proportions were selected based on previous tests, prioritizing stability and reproducibility.

The film was subsequently compressed by movable barriers at a rate of 10 mm/min. During compression, the surface pressure and surface potential were simultaneously measured as the area decreased using a Wilhelmy sensor and a potential probe, respectively. The surface pressure sensor was a filter paper plate with dimensions of 1 cm × 2 cm. Compression continued until the monolayer collapsed, and the resulting curves of surface pressure versus molecular area (π–A isotherms) and surface potential versus molecular area (Δ*V*-A isotherms) were plotted.

In dilatational rheology experiments, the monolayers were first compressed to 30 mN/m and stabilized for 10 min, during which the barriers were adjusted to maintain a steady state. The monolayer then underwent ten cycles of compression and expansion with a 1% area variation at a frequency of 20 mHz. The complex surface dilatational modulus (E*) was calculated using the formula $E^{*}=-A{\left(\frac{\Delta \pi }{\Delta A}\right)}_{T}$ with the values averaged over ten cycles. The phase shift (φ) between the sinusoidal waves representing surface pressure (stress) and area (strain) was measured, allowing for the calculation of the elastic (storage) modulus (*E′* = E* sin φ) and the viscous (loss) modulus (*E″* = E* cos φ). These experiments provided detailed insights into the monolayer’s mechanical properties, specifically its dilatational modulus and viscosity.

Brewster angle microscopy (BAM) and polarization modulation infrared reflection absorption spectroscopy (PM-IRRAS), both from KSV-Nima Instruments, were utilized to obtain reflectivity images (BAM) and infrared spectra (PM-IRRAS) from the air–water interface. The films were compressed to the desired surface pressure values, and images and spectra were collected at a constant surface pressure. PM-IRRAS spectra were obtained with an incidence angle of 80° relative to the plane normal. For the pieces of equipment, we employed a PMI-550 PM-IRRAS, both from KSV Instrument.

For transferring the floating monolayers to solid substrates, quartz supports (1 × 2 cm) were pretreated by bathing in a 0.01 M KOH solution in ethanol and sonicated for 10 min at room temperature. After being rinsed with pure water, the supports were mounted in a dipper device positioned above the air–water interface. For the LB film transfer process, the supports were initially immersed in an aqueous subphase. After the amphiphiles were spread and solvent evaporation was allowed to occur, the monolayer was compressed to a surface pressure of 30 mN/m, which was maintained by adjusting the lateral barriers. The substrate was then repeatedly withdrawn from and reimmersed in the subphase at a rate of 10 mm/s. Between the upstroke and downstroke, the substrate was allowed to dry for 10 min. Each vertical passage through the Langmuir film transferred one monomolecular layer to the solid support, with successful transfer confirmed by the transfer ratio, calculated by the ratio between the decreased monolayer area to the support area in contact with the interface during its passage. Transfer ratios close to unity indicated a uniform transfer. LB films of up to 29 monolayers were produced, with positive transfer ratios confirmed on quartz substrates. The molar ratios of the Eu:PNM2 LB films were (1:2), (1.2:1), and (3:1).

The LB films were characterized by using PM-IRRAS and photoluminescence spectroscopy. Infrared spectra were recorded by positioning the LB film under an incident beam at 76° relative to the normal. Photoluminescence spectra were obtained using a Horiba Fluorolog 3 spectrofluorimeter model FL3C-22 equipped with a Hamamatsu R928 photomultiplier and a 450 W xenon lamp. The front-face acquisition mode was used with the room temperature excitation spectra of the PNM2 + Eu films monitored at 615 nm and emission spectra recorded at an excitation wavelength of 351 and 394 nm, scanning between 570 and 720 nm. The lifetime measurements were acquired by using a phosphorimeter with a pulsed Xe–flash tube.

## Results and Discussion

Figure 2 illustrates the tensiometric response of the PNM2 monolayer compressed at the air–water interface.

**Figure 2:**
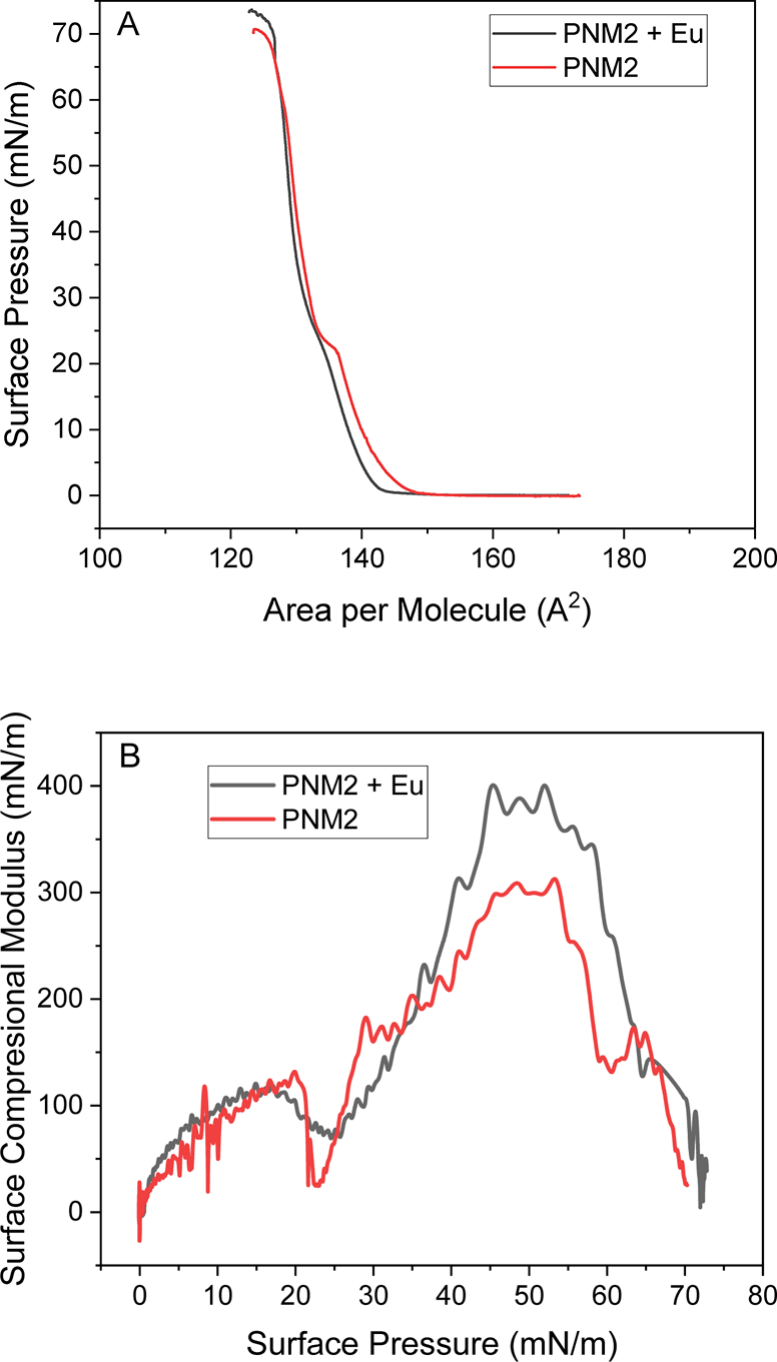
Surface pressure–area, π–*A* (A), and surface compression modulus–surface pressure, *K*–π (B), isotherms for PNM2 without or with Eu complex Eu–PNM2 molar ratio (3:1). For both solutions at the same concentration: 0.5 mg/mL.

Figure 2 panel A shows a sudden increase in surface pressure when the molecular area is compressed below 140 Å^2^, displaying a distinct shoulder around 18 mN/m, which marks the onset of a region with lower compressibility, indicating a more condensed state. The monolayer ultimately collapsed around 70 mN/m. The molecular areas observed are consistent with the expected size of PNM2 and align well with similar alkylated nucleotides.^24,25^ Upon incorporation of the europium complex, we observe an expansion of the monolayer prior to the shoulder, with the width of this expansion increasing relative to the isotherm for the pure prodrug. This is the first indication of complex incorporation. After the shoulder, the isotherms converge, suggesting the high compactivity of the film. While one hypothesis is that the complex may be expelled toward the air phase, causing aggregation, further microscopy measurements and transfer to solid supports at high pressures refute this possibility.

Using the formula proposed by Davies and Rideal:^26^ $K=-A{\left(\frac{\partial \pi }{\partial A}\right)}_{T}$ , the K values for PNM2 alone reach as high as 400 mN/m (Figure 2, panel B), indicating a transition between the liquid-condensed state and the solid. In contrast, the presence of the europium complex reduces the *K* values to approximately 300 mN/m, suggesting a less condensed state. Both curves exhibit a trough around 20 mN/m, corresponding to the shoulder in the isotherms. The introduction of europium complexes into the rigid PNM2 monolayers decreases the surface compression modulus through several interconnected mechanisms. The bulky nature of these complexes disrupts the tight packing of lipid molecules, increasing spacing and reducing overall density. This disruption also enhances the flexibility of the PNM2 chains, rendering the structure less rigid and more compressible. Additionally, if the europium complexes possess amphiphilic characteristics, they may modify the orientation and arrangement of the lipid molecules, impacting cohesive forces within the monolayer. Electrostatic interactions, due to the charged nature of the complex, can further alter the organization of lipid head groups, while changes in hydration dynamics around the lipids may also contribute. Collectively, these factors lead to a significant reduction in the effective compressive strength of the monolayer.

The monolayer was also subjected to two cycles of compression–expansion (with returning surface pressure of 30 mN/m), and we did not observe significant hysteresis, indicating reversibility of the system Supporting Information (Figure S1). It means that the molecular arrangement and interactions within the film remain stable throughout the cycling of pressure. This absence of hysteresis suggests that the surfactant molecules are uniformly distributed and can easily rearrange themselves without forming permanent structures or aggregates. As a result, upon re-expansion, the monolayer can return to its original state without significant changes in molecular organization or properties, reflecting a strong compatibility and favorable interactions among the molecules in the monolayer. This behavior is typically associated with ideal monolayer systems where intermolecular forces are balanced and the film maintains its integrity under varying pressures.^27^ Another indication that no aggregation occurred is the absence of interfacial domains, as observed through BAM. The microscopy reveals a homogeneous monolayer of PNM2, both with and without the europium complex, as shown in the Supporting Information (Figure S2).

When the monolayer is compressed to 30 mN/m and subjected to short cycles of compression and expansion (1% in area), dilatational rheological properties can be obtained. The so-called dilatational surface is mathematically corresponding to K; however, the surface compression modulus quantifies the resistance of the monolayer to changes in area under uniform compression, reflecting how much the surface pressure increases when the area is decreased. In contrast, dilatational surface elasticity measures the monolayer’s response to changes in area under nonuniform conditions, such as when the surface is expanded or contracted at a specific point. While the compression modulus focuses on compressive stresses, dilatational elasticity accounts for both the compressive and tensile responses, providing insights into the film’s behavior during dynamic processes like expansion and contraction, and the possible effect of the surface viscosity of the monolayer.^28^ As a result, Table 1 presents the data obtained from oscillations near 30 mN/m, as compared to those reported previously.^17^ Oscillation data are shown in Supporting Information (Figure S3).

**Table 1:** dilatational Surface Elasticity for PNM2 and PNM2+Eu Complex, with Oscillation Starting at 30 mN/m (1% in Area and 20 mHz) at (3:1) Eu:PNM2 Molar Ratio for Both Solutions at the Same Concentration: 0.5 mg/mL

monolayer	E* (mN/m)	*E*′ (mN/m)	*E*″ (mN/m)	φ (rad)
PNM2	263.3	262.5	103.7	0.38
PNM2+Eu	1010.1	995.1	–184.6	–1.39

Notably, when the europium complex is incorporated, the elasticity value is relatively high compared to the K values. This behavior appears to result from the stability cycle at 30 mN/m before the oscillation process begins, coupled with the molecular accommodation provided by the oscillation cycles within the supramolecular system containing the complex. As a result, the monolayer remains rigid, preventing fluidization, as indicated by uniaxial compression. This rigidity is crucial for transferring the monolayer to solid supports such as Langmuir–Blodgett (LB) films, where monolayer stability is essential for achieving effective molecular transfer. Rigid monolayers are more likely to be displaced from the fluid interface to the solid surface, facilitating a better transfer process.^29^

The negative values of *E*″ imply negative viscosity. Although this lacks physical significance, it is frequently reported for Langmuir monolayers and is identified when the monolayer exhibits a counterintuitive response to deformation, where instead of resisting compression and expansion, it actually accelerates these processes. This phenomenon occurs due to specific molecular interactions within the monolayer, leading to active or unstable behavior. During the experiments, such a response is detected by observing an out-of-phase component in the stress–strain relationship, indicating that the monolayer’s surface stress decreases as it is compressed, which is characteristic of negative viscosity.^30^ We believe that this behavior should be associated with the molecular accommodation of europium complex going to a less rigid state, as observed in the surface compression modulus data, to a more rigid one, as observed in the absolute values of E*.

The surface potential-area isotherms (Figure 3) demonstrate the changes in the electric environment upon the incorporation of europium complexes. The isotherm for pristine PNM2 was previously reported^18^ and presents a maximum surface potential of 400 mV, which is attributed to the organization of its electric dipoles in an orthogonal orientation relative to the air–water interface when fully compressed.^31^ The introduction of the europium complex lowers the initial surface potential values to negative, likely due to the presence of poorly distributed charges in the fluid phase (aqueous subphase) that are weakly adsorbed or situated near the charged environment at the polar region, resulting in the formation of a double-charged layer.^32^ This finding confirms the incorporation of the charged europium complex, even at low molecular densities. When the hybrid monolayer is compressed, the maximum surface potential values approach those of pure PNM2, indicating the maintenance of a rigid structure with the organized electric dipoles, now with minimal influence from the electric double layer.

**Figure 3:**
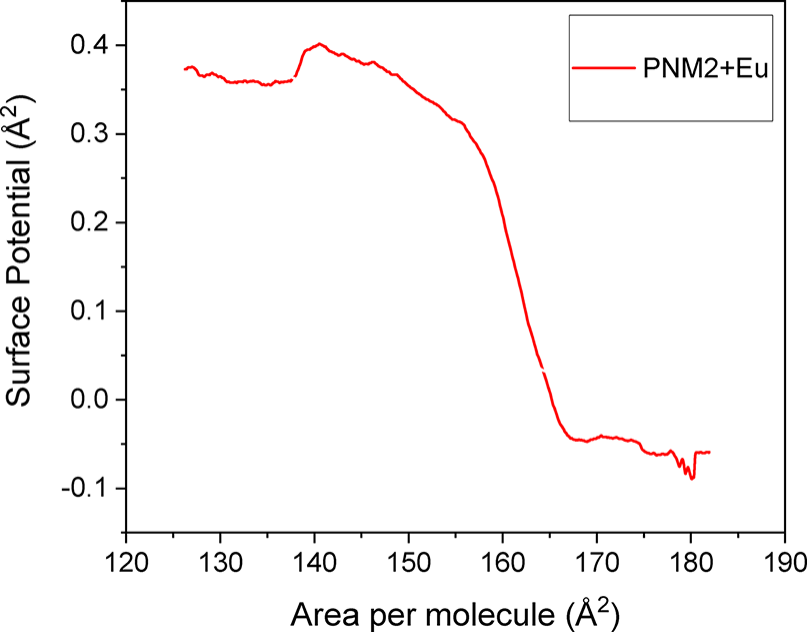
Surface potential–area, Δ*V*–*A*, isotherms for PNM2 with Eu complex (3:1) Eu–PNM2 molar ratio for both solutions at the same concentration: 0.5 mg/mL.

The monolayers were then transferred to solid supports, and the transfer ratio values gave values close to unity, confirming the uniformity of the transfer. The LB films were first characterized with PM-IRRAS, as shown in Figure 4.

**Figure 4:**
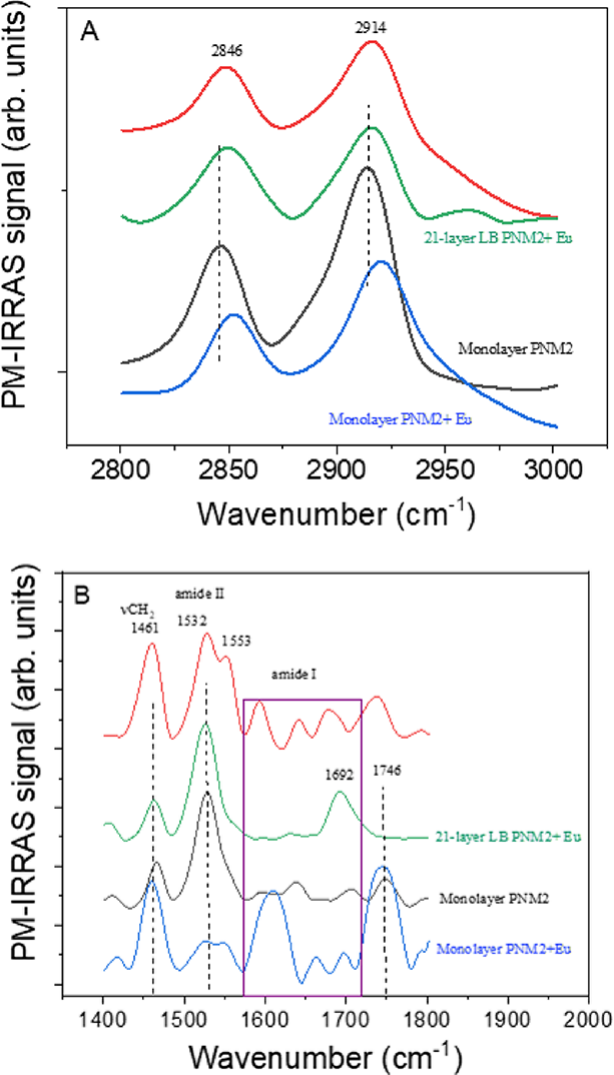
PM-IRRAS for PNM2 films without or with Eu complex Eu–PNM2 (3:1) molar ratio for both solutions at the same concentration: 0.5 mg/mL: (A) CH_2_ and (B) C=O region.

Figure 4 panel A shows the bands centered at 2846 and 2914 cm^–^^1^ in the floating monolayers attributed to CH_2_ stretches (symmetric and asymmetric, respectively). Low wavenumbers indicate a high degree of molecular organization, suggesting a high all-trans/gauche ratio.^33^ When the europium complex is introduced, these bands shift to higher wavenumbers, consistent with the compression modulus data, indicating a less rigid monolayer. This reduced rigidity likely results from the alkyl chains of PNM2 being less tilted relative to the air–water interface, leading to a less densely packed structure with voids in the supramolecular arrangement. Upon transfer to solid supports as LB films, this structural organization is maintained, confirming the preservation of packing on the solid support.

The C=O stretching bands (Amide I and ester) are shown in Figure 4, panel B. These bands are highly sensitive to changes in the molecular environment,^34^ and we observed significant alterations in both their relative intensities and peak positions. These changes suggest an interaction between the europium complex and carbonyl groups. Furthermore, the extent of these changes varies depending on the support medium, whether water or solid.

The europium ion Eu^3+^ was used as a structural probe, providing detailed information about the local site symmetry. Figure 5 presents the excitation spectra of the LB–Eu–PNM2 films with varying molar ratios (3:1, 1.2:1, and 1:2) monitored at the hypersensitive ^5^D_0_ → ^7^F_2_ transition at 615 nm. The broad bands observed at higher energies are attributed to excited states of the ligand.^35^ The observed shifting and broadening of these bands suggest interactions between the europium complex and PNM2. Furthermore, the low intensity of the ^7^F_0_ → ^5^L_6_ intra f–f transitions, compared to the Eu­(tta)_3_(H_2_O)_2_, implies effective sensitization of the europium ion upon incorporation into PNM2. In addition, at higher europium concentration (3:1), an increase in the intensity of the ^7^F_0_ → ^5^L_6_ transition is observed, suggesting that sensitization also occurs through the Eu^3+^.

**Figure 5:**
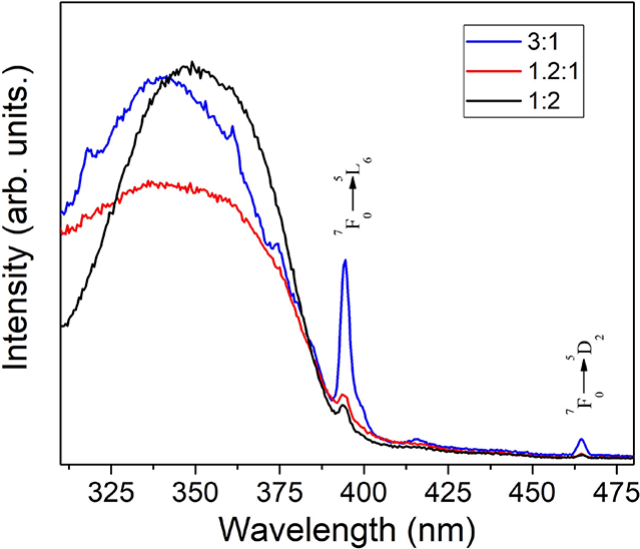
Room temperature excitation spectra monitored at 615 nm ^5^D_0_ → ^7^F_2_ for Eu–PNM2 at (3:1), (1.2:1), and (1:2) molar ratios.

Figure 6 panel A shows the emission spectra of Eu–PNM2 LB films, excited at 351 nm in the broadband of the ligand. The most intense transition observed is related to the ^5^D_0_ → ^7^F_2_ emission line, so-called hypersensitive and dependent on environment, indicating low symmetry without an inversion center of the Eu^3+^ ions. Additionally, the presence of the ^5^D_0_ → ^7^F_0_, forbidden by selection rules, indicates a *J-mixing* due to crystal field effect and that europium ions occupy point groups such as C_nv_, C_n_, or C_s_.^36^

**Figure 6:**
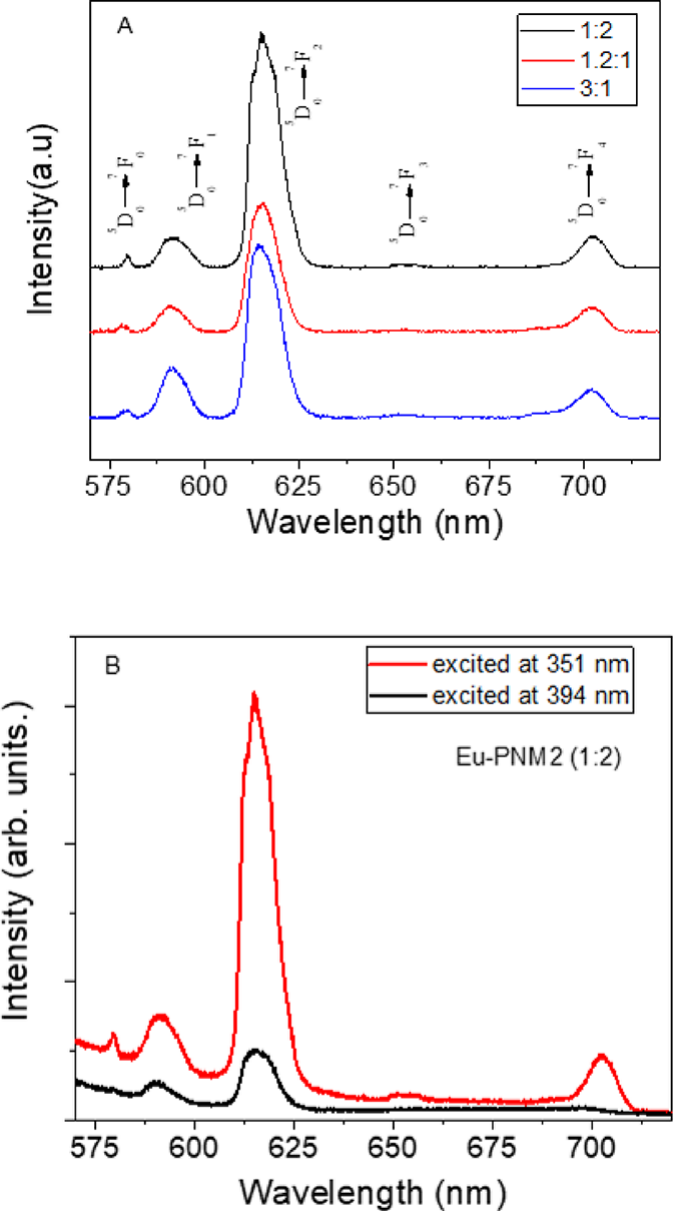
Room temperature emission spectra of the Eu–PNM2 excited at 351 nm ^5^D_0_ → ^7^F_2_ for Eu–PNM2 for 3:1, 1.2:1, and 1:2 LB films (A) and emission spectra of the Eu–PNM2 excited at 351 and 394 nm for 1:2 LB film (B).

To verify the antenna effect with which the ligand transfers energy to the Eu^3+^, the film Eu–PNM2 (1:2) was excited at 351 nm in the broad band of the ligand and at 394 nm directly in the Eu^3+^ ion. Figure 6 panel B depicts the emission spectra, which clearly show a higher intensity of the ^5^D_0_ → ^7^F_2_ emission line when excitation occurs through the ligand broad band.

Figure 7 shows the experimental lifetime (τ_exp_) values obtained through the monitoring decay curves at 615 and excited at 351 nm by fitting a mono-exponential function. The values obtained from 0.48 to 0.63 ms are higher than those reported for the pristine complex of approximately 0.3 ms,^37^ indicating that the concentration of complex and interaction between Eu–PNM2 contribute to increasing the radiative process.

**Figure 7:**
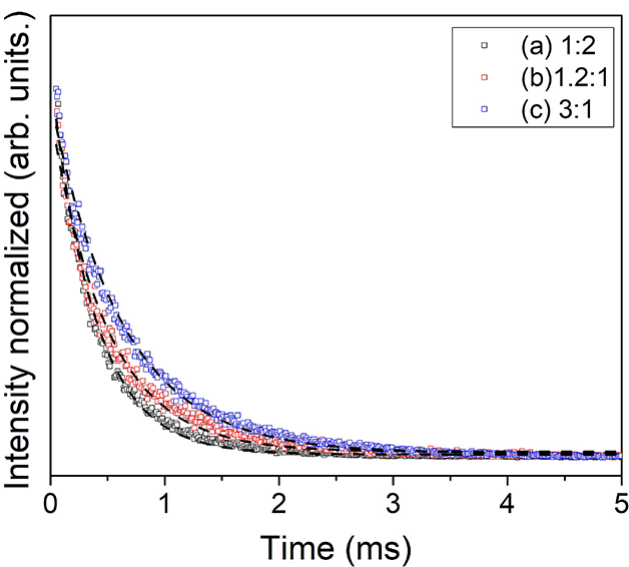
Decay curves for Eu–PNM2 LB films at (a) 1:2, (b) 1.2:1, and (c) 3:1 molar ratios excited at 351 nm and emission at 615 nm.

The quantum efficiency (*q*) was obtained using eqs 1 and 2, which consider both radiative and nonradiative depopulation processes of the emitting ^5^D_0_ level.^38^

$$q\;(\%)=\frac{A_{\mathrm{rad}}}{A_{\mathrm{rad}}+A_{\mathrm{nrad}}}$$ 1

$$A_{\mathrm{total}}=A_{\mathrm{rad}}+A_{\mathrm{nrad}}=\frac{1}{\tau_{\exp}}$$ 2

The symmetry of Eu^3+^ was assessed by the relative intensity ratio of the transitions ^5^D_0_ → ^7^F_2_ and ^5^D_0_ → ^7^F_1_, denoted as (*I*_0–2_/*I*_0–1_). A decrease in this ratio with an increasing complex content (3:1) indicates enhanced symmetry around the Eu^3+^ ion.

The number of water molecules (*n*_w_) coordinated to the Eu^3+^ ion was estimated using Horrocks’ equation 3.^39^

$$n_{H2O}=1.11\times (A_{\exp}-A_{\mathrm{rad}}-0.31)$$ 3

The pristine complex [Eu­(tta)_3_(H_2_O)_2_] exhibits two molecules of water in its coordination sphere. Upon incorporation into the LB film of a higher concentration of complex Eu–PNM2 (3:1), the estimated number of coordinated water molecules decreases to one. This reduction suggests a decrease in the nonradiative decay pathway due to O–H oscillators.^40^ This behavior implies that one molecule of water is replaced by the interaction of the oxygen atoms of the C=O groups from PNM2, as evidenced by notable changes in the Amide I and II regions observed in the PM-IRRAS spectra. Furthermore, Judd–Ofelt intensity parameters Ω_2_ and Ω_4_ were determined from the emission spectra.^41^ At higher concentrations of the complex with a 3:1 molar ratio, the Ω_2_ values decrease, indicating changes in the local environment of the europium ion, as well as a reduction in the covalency of the Eu^3^^+^–oxygen bonds within the PNM2 molecular structure. This behavior is consistent with a decrease in the number of coordinated water molecules, which leads to an increase in the experimental luminescence lifetime and promotes radiative decay pathways. Additionally, an increase in local symmetry is observed, as indicated by the (*I*_0–__2_/*I*_0–__1_) intensity ratio as a function of the complex concentration. Moreover, the low values of the Ω_4_ parameter suggest a more rigid Eu–PNM2 coordination environment and imply that the ligand framework remains structurally preserved. Table 2 summarizes the *A*_rad_/s^–1^, *A*_nrad_/s^–1^, τ_exp_, *q*(%), η­(w), *I*_0–2_/*I*_0–1_, Ω_2_, and Ω_4_ values.

**Table 2:** Radiative (*A*_rad_) and Nonradiative (*A*_nrad_) Decay Rates, Experimental Lifetimes (τ_exp_), Quantum Efficiency (*q*%), Number of Water Molecules (η­(w), Relative Intensities Ratio (*I*_0–2_/*I*_0–1_), and Judd–Ofelt Parameters (Ω_2_ and Ω_4_)

LB films Eu–PNM2	*A*_rad_/s^–1^	*A*_nrad_/s^–1^	τ_exp_ (ms)	*q* (%)	η (w)	*I*_0–2_/*I*_0–1_	Ω_2_/10^–20^ (cm^2^)	Ω_4_/10^–20^ (cm^2^)
3:1	333	1151	0.63	21	1.05	4.4	7.7	2.1
1.2:1	456	1002	0.49	22	1.41	6.0	10.0	3.6
1:2	567	958	0.38	22	1.95	8.3	15.0	3.1

Figure 8 shows the Commission International de L’Éclairage (CIE) diagram indicating the coordinates (*x* = 0.66 and *y* = 0.34) obtained from the emission spectra of the Eu–PNM2 (3:1) film. The black triangle indicates the color position in the diagram.

**Figure 8:**
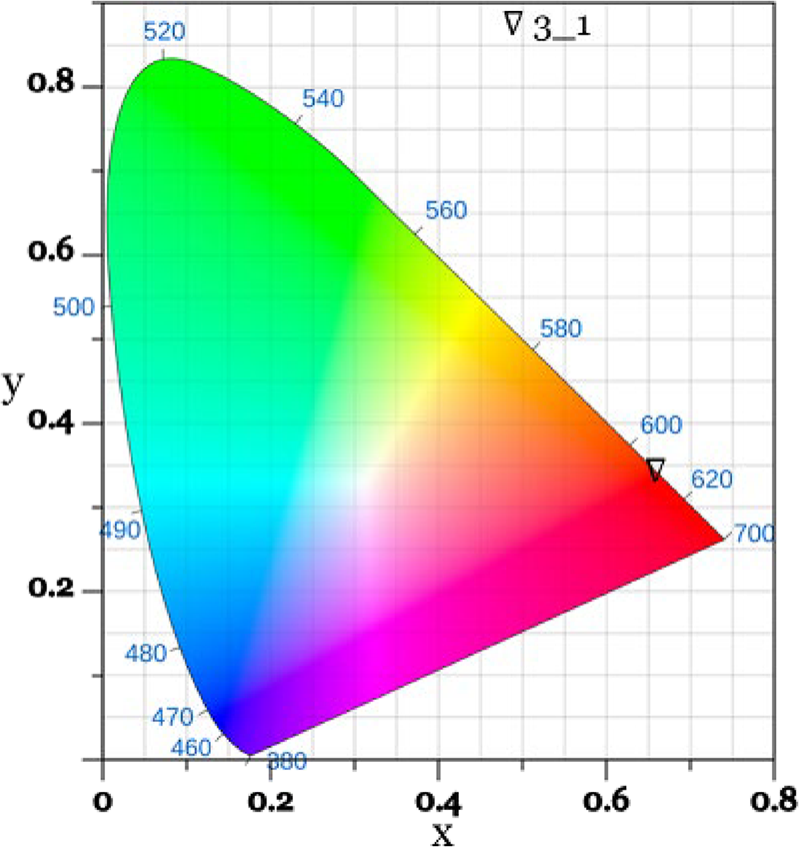
Chromaticity diagram (CIE) presenting (*x*, *y*) coordinates (0.66 and 0.34) of the Eu–PNM2 LB film excited at 351 nm.

Finally, compared to more commonly used platforms such as liposomes, polymeric nanoparticles, or self-assembled monolayers,^42–44^ LB films offer several distinct advantages for the systematic design and investigation of nanostructured biointerfaces. They allow precise control over film thickness, composition, and molecular orientation at the nanometer scale—features that are particularly valuable when studying interactions between therapeutic agents, such as PNM2, and biomolecular targets or when optimizing energy transfer in luminescent systems. In addition, LB films provide planar, two-dimensional architectures that closely mimic biological membranes, unlike bulk systems such as liposomes or polymeric carriers. This facilitates the investigation of supramolecular organization and controlled drug–lipid or drug–protein interactions in a bioinspired setting.

Their compatibility with solid supports such as quartz enables direct coupling with advanced spectroscopic and microscopic techniques, including PM-IRRAS, BAM, and photoluminescence spectroscopy—tools that are less readily applicable in colloidal systems. This compatibility was essential for characterizing the antenna effect and enhanced luminescence lifetimes observed in the Eu–PNM2 system. Furthermore, LB films exhibit good physical stability and can be stored or reused for repeated measurements, which is not always feasible with dispersions such as micelles or vesicles that are more prone to aggregation or degradation.

Nonetheless, it is important to acknowledge that LB films are best suited for surface-bound applications and in vitro or ex vivo diagnostics. In contrast, platforms such as polymeric nanoparticles are more appropriate for systemic in vivo delivery. Therefore, our current approach focuses on exploiting LB films as biofunctional diagnostic surfaces or localized drug delivery platforms, for example, in implantable coatings or optical biosensors, rather than as carriers for systemic therapeutic administration.

## Conclusion

In this study, we successfully developed and characterized Langmuir and LB films composed of a uridine ester derivative (PNM2) and the europium complex Eu­(tta)_3_(H_2_O)_2_, as a preliminary step toward the development of theranostic platforms for cancer research. The PNM2 monolayers exhibited high rigidity and molecular ordering, which were modulated upon incorporation of the europium complex, leading to increased molecular spacing and a transition to a more fluid monolayer, as confirmed by surface compression modulus analysis and PM-IRRAS spectral shifts. Despite this fluidization, the monolayers retained sufficient structural integrity to be transferred as homogeneous LB films, as evidenced by high transfer ratios and morphological uniformity. Rheological data further demonstrated enhanced dynamic elasticity in the presence of the europium complex, indicating robust intermolecular interactions. Photoluminescence analyses showed that the Eu^3^^+^ complex maintained its characteristic emission properties within the LB films, with pronounced antenna effects and environmental sensitivity, confirming efficient energy transfer from the tta ligand. Spectroscopic data revealed reduced water coordination and increased local symmetry around Eu^3^^+^, consistent with interactions involving the PNM2 carbonyl groups. Taken together, these results demonstrate that PNM2–Eu­(tta)_3_(H_2_O)_2_ LB films exhibit structural stability, optical responsiveness, and molecular specificity, supporting their potential as biofunctional surfaces for future theranostic applications. While this study focuses on physicochemical and spectroscopic characterization, it lays the groundwork for subsequent investigations into biological performance such as drug delivery, cellular interaction, and cytotoxicity.

## Supplementary Material



## Notes

Compression–Expansion curves, BAM images, and crude data of the monolayer area oscillation are available in Supporting Information file (PDF)
