# Supplementary material for: Design and Characterization of Langmuir–Blodgett Films Incorporating Europium Complexes and Nucleoside Derivatives for Cancer Therapeutic Applications
Source: ACS Omega. 2025 Sep 12;10(37):43061–8. doi: 10.1021/acsomega.5c06204 (PMC12461355; doi:10.1021/acsomega.5c06204)
Supplement: Supplementary file 1 [file ao5c06204_si_001.pdf]

Suplementary Materials

Design and Characterization of Langmuir-Blodgett Films Incorporating Europium Complexes and Nucleoside Derivatives for Cancer Therapeutic Applications

Guilherme Coutinho Pereira[1], Kevin Figueiredo dos Santos[1], Jhon Fernando Berrío Escobar[3,4] , Cristiano Giordani[2,3], Luciano Caseli[1*], and Celso Molina[1]

[1]Department of Chemistry, Federal University of São Paulo, Diadema, SP, Brazil

[2] Institute of Physics, Faculty of Exact and Natural Sciences, Universidad de Antioquia, UdeA, Calle 70 No. 52-21, Medellín 050010, Colombia.

[3]Grupo Productos Naturales Marinos, Faculty of Pharmaceutical and Food Sciences, Universidad de Antioquia, UdeA, Calle 70 No. 52-21, Medellín 050010, Colombia.

[4] Institución Educativa Ciudad Dorada-Secretaría de Educación de Armenia - Colombia

* lcaseli@unifesp.br

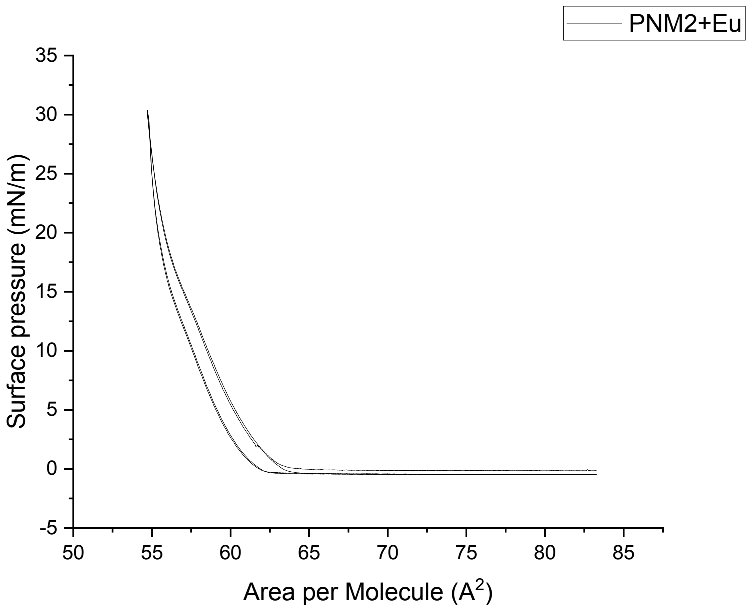

Figure S1: Compression-Expansion isotherms for PNM2 (two cycles) with Eu complex Eu-PNM2 molar ratio (3:1). For both solution at the same concentration: 0.5 mg/mL)

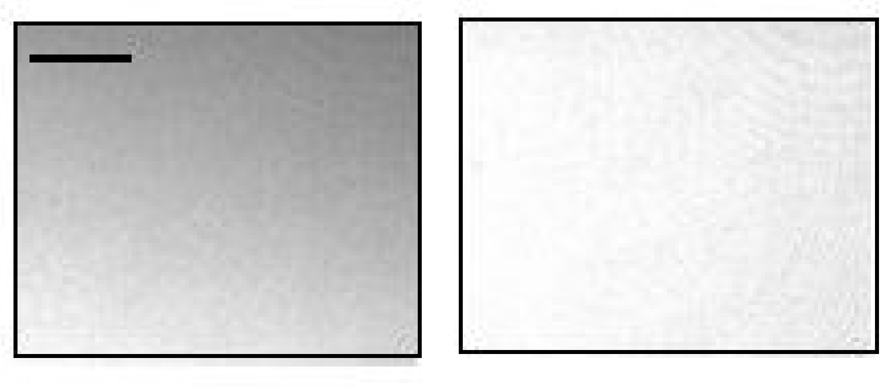

Figure S2: BAM images (scale bar: 100 mm) for PNM2 monolayers at 30 mN/m without (left) or with Eu complex Eu-PNM2 molar ratio (3:1). For both solution at the same concentration: 0.5 mg/mL).

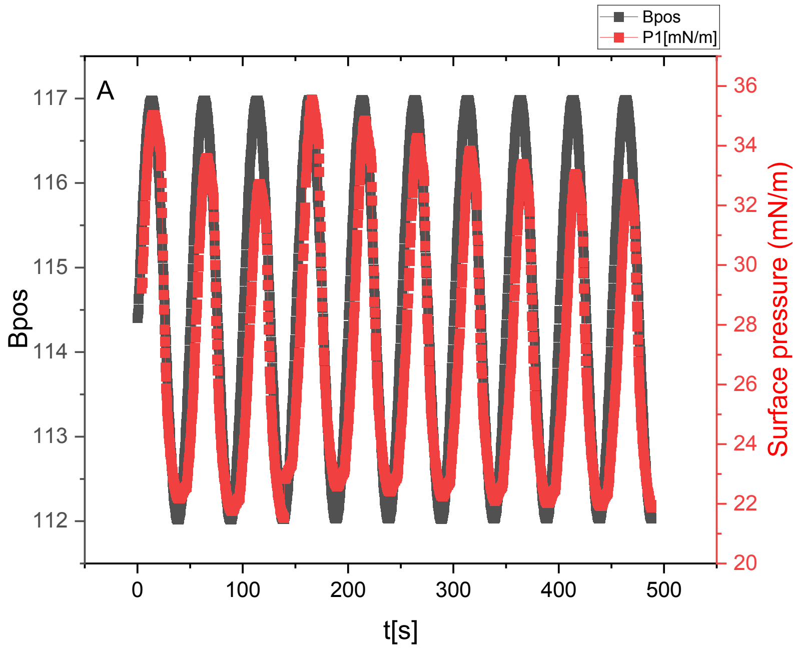

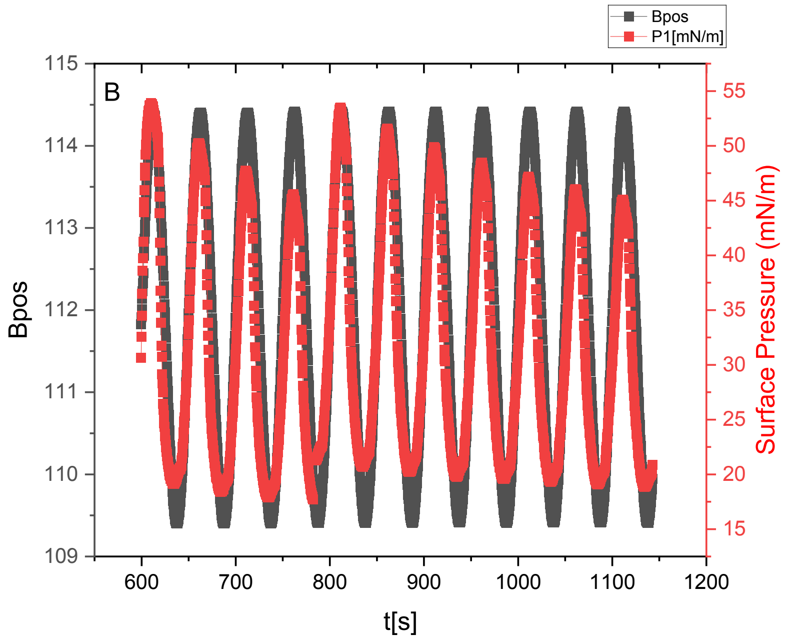

Figure S3: Oscillation experiments for PNM2 monolayers at 30 mN/m without (A) or with Eu complex Eu-PNM2 molar ratio (3:1) (B). For both solution at the same concentration: 0.5 mg/mL, frequency of 20 mHz and 1% of area oscillation.
